# *Plasmodium vivaxmdr1* genotypes in isolates from successfully cured patients living in endemic and non-endemic Brazilian areas

**DOI:** 10.1186/s12936-016-1141-9

**Published:** 2016-02-18

**Authors:** Larissa Rodrigues Gomes, Natália Ketrin Almeida-de-Oliveira, Aline Rosa de Lavigne, Suelen Rezende Félix de Lima, Anielle de Pina-Costa, Patrícia Brasil, Cláudio Tadeu Daniel-Ribeiro, Didier Ménard, Maria de Fatima Ferreira-da-Cruz

**Affiliations:** Laboratório de Pesquisa em Malária - Instituto Oswaldo Cruz, Fundação Oswaldo Cruz (Fiocruz), Rio de Janeiro, Brazil; Centro de Pesquisa, Diagnóstico e Treinamento em Malária (CPD-Mal) Fiocruz, Rio de Janeiro, Brazil; Laboratório de Doenças Febris Agudas - Instituto Nacional de Infectologia Evandro Chagas (INI-IPEC) (Fiocruz), Rio de Janeiro, Brazil; Malaria Molecular Epidemiology Unit, Institut Pasteur in Cambodia, Phnom Penh, Cambodia

**Keywords:** *Plasmodium vivax*, Chloroquine resistance, *pvmdr1* gene, Brazil

## Abstract

**Background:**

*Plasmodium vivax* is the most widely distributed species causing the highest number of malaria cases in the world. In Brazil, *P. vivax* is responsible for approximately 84 % of reported cases. In the absence of a vaccine, control strategies are based on the management of cases through rapid diagnosis and adequate treatment, in addition to vector control measures. The approaches used to investigate *P. vivax* resistance to chloroquine (CQ) were exclusively in vivo studies because of the difficulty in keeping parasites in continuous in vitro culture. In view of the limitations related to follow-up of patients and to assessing the plasma dosage of CQ and its metabolites, an alternative approach to monitor chemo-resistance (QR) is to use molecular markers. Single nucleotide polymorphisms (SNPs) in the multidrug resistance gene *pvmdr1* are putative determinants of CQ resistance (CQR), but such SNPs in *P. vivax* isolates from patients with good response to treatment should be further explored. The aim of this study is to investigate the mutations in the gene, supposedly associated to QR, in *P. vivax* isolates from successfully cured patients, living in Brazilian endemic and non-endemic areas.

**Methods:**

Blood samples were collected from 49 vivax malaria patients from endemic (Amazon Basin: 45) and non-endemic (Atlantic Forest: four) Brazilian regions and analysed for SNPs in the CQR-related *P. vivax* gene (*pvmdr1*), using PCR-based methods.

**Results:**

Among the 49 isolates genetically characterized for the gene *pvmdr1*, 34 (70 %) presented at least one mutation. T958**M** mutant alleles were the most frequent (73 %) followed Y976**F** (15 %) and F1076**L** (12 %). Single mutation was detected in 24 (70.5 %) isolates and double mutations in ten (29.5 %). The most common single mutant genotype was the 958**M/**Y976/F1076 (79 %), followed by 976**F/**F1076 (21 %) whereas 958**M/**Y976/1076**L** (60 %) and 976**F/**1076**L** (40 %) double mutant genotypes were detected. Single mutant profile was observed only in isolates from Amazon Basin, although double mutants were found both in the Amazon and Atlantic Forest regions. Interestingly, the genotype 958**M**/Y976/1076**L** was present in all isolates from the Atlantic Forest in the Rio de Janeiro State.

**Conclusions:**

Considering that primaquine (PQ) efficacy is highly dependent on concurrent administration of a blood schizontocidal agent and that PQ could not circumvent CQR, together with the fact that no *pvmdr1* mutation should be expected in successfully cured patients, these findings seem to indicate that the *pvmdr1* gene is not a reliable marker of CQR. Further investigations are needed to define a reliable molecular marker for monitoring *P. vivax* CQR in *P. vivax* populations.

## Background

Almost 40 % (approximately three billion) of the world’s population is presently at risk of contracting malaria. The disease causes almost 200 million clinical cases and around 600,000 deaths each year [1, 2]. *Plasmodium vivax* is the most geographically widespread of the human malaria parasites, and a serious public health concern in South and Central America, Asia and Southwest Pacific [3].

In Brazil, endemic regions are restricted to the Legal Amazon (comprising Acre, Amapá, Amazonas, part of Maranhão, Mato Grosso, Pará, Rondonia, Roraima and Tocantis States), a region that presently accounts for 99.6 % of the countrywide malaria burden [4]. *Plasmodium vivax* is the predominant species, responsible for 84 % of the reported cases [5], PNCM, SVS, MS, unpublished data 2015. Extra-Amazonian autochthonous cases account for only 0.04 % of all Brazilian total registered and correspond to the autochthonous malaria existing in the Atlantic Forest, located along the southeastern Atlantic Coast [6].

Chloroquine resistance (CQR) is the main challenge for national malaria control programmes to control vivax malaria. The first cases of *P. vivax* resistant to chloroquine (CQ) were described in Papua New Guinea [7] and thereafter observed in Indonesia [8], Oceania, Asian [9, 10] and South American countries, including Brazil [11, 12]. In Brazil, CQ treatment failures, presumably related to CQR, have been reported [13]. The latest 28 day in vivo test conducted to assess the efficacy of standard supervised CQ therapy in 109 volunteers showed a proportion of 10.1 % of treatment failure (n = 11), despite an adequate absorption of CQ in these individuals on day 2 [14].

Molecular markers can represent a valuable tool for monitoring introduction and spread of drug resistance. Contrarily to *Plasmodium falciparum*, mutations at codons in the *pfcrt* orthologue (*pvcg10*) gene do not seem to mediate CQR in *P. vivax* [15]. On the other hand, the polymorphisms at codons Y976**F** and F1076**L** in the multidrug-resistant gene 1 (*pvmdr1*) has been described as molecular marker associated to CQR [16]. Indeed, in Thailand, Indonesia [17] and Myanmar [18], as well as in Mauritania [19] and Cambodia [20], it has been shown that 976**F** mutants were associated with clinical resistance to CQ. In Nepal and India, where *P. vivax* CQR has not been recorded, prevalence of the 976**F** mutation is very low (5 %) [21] or not detected [22], while in India the presence of the F1076**L** mutation was not associated to CQR. In addition, in Madagascar, despite 5 % of clinical failures more than 90 % of Y976**F** mutant parasites were detected [33].

These polymorphisms also seem to be relatively uncommon in Latin America, where *P. vivax* CQR remains relatively infrequent [23]. In Brazil, different conclusions were drawn: either mutations in *pvmdr1* were reported in CQ-sensitive *P. vivax* parasites [24–26] or not detected in resistant *P. vivax* isolates [25, 26], as well as *P. vivax* CQR being associated with *pvmdr1* mutants only in patients with severe malaria [27].

In view of these different epidemiological data, the nucleotide polymorphisms (SNPs) of *pvmdr1* gene in successfully cured vivax malaria patients living in endemic (Amazonian) and non-endemic (Extra-Amazonian) Brazilian areas, were investigated in the present study.

## Methods

### Study site, blood samples and DNA extraction

Blood samples were collected between 2010 and 2014 in patients presenting vivax malaria (n = 49) at the Laboratório de Doenças Febris Agudas, INI-IPEC, Fiocruz, the Reference Laboratory for Malaria in the Extra-Amazon to the Brazilian Ministry of Health. The inclusion criterion was patients with uncomplicated vivax malaria. After obtaining informed consent, venous blood collection was performed according to protocols previously approved by the Ethical Research Committees of Fiocruz (32839013.6.00005248). Genomic DNA was extracted from 1 mL whole blood using QIAamp midi columns, as described by the manufacturer (Qiagen). *Plasmodium vivax* samples were diagnosed by microscopic examination and by polymerase chain reaction (PCR) [28]. All patients were treated with CQ plus primaquine (PQ) and followed up for 42 days and no treatment failure was detected during this period.

### PCR and electrophoresis

The *pvmdr1* gene was amplified by PCR using gene-specific primers. The PCR was performed as described elsewhere [16, 29] to amplify a partial DNA sequence containing three SNPs for *pvmdr1* gene including: T958**M**, Y976**F** and F1076**L**.

### DNA sequencing and SNP polymorphisms detection

After purification using the Wizard SV Gel and PCR Clean-Up System (Promega), the amplified fragments were sequenced using Big Dye^®^ Terminator Cycle Sequencing Ready Reaction version 3.1 (Applied Biosystems) and ABI PRISM DNA Analyzer 3730 (Applied Biosystems) [30] at the Genomic Platform/PDTIS/Fiocruz. The direct DNA sequencing from PCR products was compared with the reference wild type Sal I GenBank accession nº AY571984 [24, 25]. Forward and reverse sequences were analysed using the free software Bioedit Sequence Alignment Editor version 7.2.5. Statistical significance of differences of *pvmdr1* genotypes frequencies among Brazilian localities was assessed using Fisher’s tests.

## Results

The *pvmdr1* gene was successfully amplified and DNA sequenced in 49 isolates from the Amazon Region (Acre, Amazonas, Pará, and Rondonia) and the Extra-Amazonian State of Rio de Janeiro.

Globally, 34 (69 %) showed non-synonymous (958**M**, 976**F** and 1076**L**) mutations. 958**M** mutant alleles were the more frequent (25/34; 73 %) while 976**F** (5/34; 15 %) and 1076**L** (10/34; 12 %) were detected at lower frequencies (Table 1). Single mutation was observed in 24 isolates (70.5 %, 24/34), while double mutations were recorded in ten (29.5 %, 10/34) *P. vivax* samples. In the isolates presenting single mutant genotype, the **M**YF profile was predominant (19/55 %) contrasting with the **F**F, which was found in only five isolates (Table 2).

**Table 1:** Frequency of 958**M**, 976**F** and 1076**L** mutants in *pvmdr1* gene among 49 Brazilian *P. vivax* isolates

SNPs	Number of isolates (%)
No mutation	15 (31)
958**M**	25 (51)
976**F**	5 (10)
1076**L**	4 (8)

*SNPs* single nucleotide polymorphisms

**Table 2:** Proportion of the 4 alleles observed among 49 Brazilian *P. vivax* isolates, according to the sampling location

Genotypes	*Pv* isolates N (%)	Localities				
		Rondônia (n = 17)	Pará (n = 11)	Amazonas (n = 8)	Acre (n = 5)	Rio de Janeiro (n = 4)
Wt Sal1 type	15 (31)	4	5	4	2	0
Single **F**F	5 (15)	1	4	0	0	0
Double **FL**	4 (12)	2	0	2	0	0
Single **M**YF	19 (55)	10	2	5	2	0
Double **M**Y**L**	6 (18)	0	0	1	1	4
		13 (76 %)	6 (54 %)	8 (67 %)	3 (60 %)	4 (100 %)

*Pv*, *Plasmodium vivax*

The *pvmdr1* wild-type allele was prevalent in Pará (54 %) followed by Acre (40 %), Amazonas (33 %), and Rondonia (24 %) states without statistically significant difference in proportion (p > 0.05). Single mutants were observed only in isolates from the Brazilian Amazon: the **M**FY allele was prevalent in Rondonia (77 %), followed by Amazonas (62 %) and Acre (67 %) (p > 0.05), although in Pará the **F**F single mutant was more frequent (66 %) than the **M**YF (p > 0.05). However, when double mutants were investigated, samples presenting **FL** (12 %) and **M**Y**L** (20 %) in both Amazonian and Extra Amazonian States, were identified. Irrespective to the Brazilian State **FL** double mutant was the less frequent (Table 2) (p > 0.05). Interestingly, all isolates from the Extra Amazon (Rio de Janeiro State) showed double mutant genotype (**M**Y**L**) contrasting with those from the Amazon Basin (6 %) (p = 0.01), where most of the isolates came from.

## Discussion

CQ and PQ remain the drugs of choice to treat vivax malaria, but recent studies have reported *P. vivax* cases of resistance to CQ in different regions of the world [9, 31], including Brazil [11–13]. Therefore, monitoring the efficacy of CQ in the treatment of vivax malaria is essential for early warning systems to promote drug policies.

To circumvent the limitations of in vivo and in vitro studies and to assess chemo-resistance, identification of mutations in target genes has been proposed, such as those in the *pvmdr1* gene at codons 976 and 1076, as well as the increased expression of *pvcrto* transcripts [12, 32]. Similar to previous studies performed with samples from western Brazilian Amazon [24, 25], in this work the T958**M** mutant was found to be the more frequent in the Brazilian Amazon and even in isolates from the Extra Amazonian regions. Additionally, in Madagascar [33], Nepal [21] and Thailand [34], most of the samples presented mutations at 958 position, although all isolates have been obtained from individuals with successful response to CQ therapy. No later than November 2015, Schousbe and colleagues [35] reported a high prevalence of 958**M** (97.6 %) among *P. vivax* samples from six different geographical sites, suggesting that this allelic variant is most likely not associated with CQR and could be an allele characteristic of Asia and Africa isolates. The present data reinforce the lack of association of 958**M** with CQR, but are not in agreement with Asian and African geographical characteristic of this allele, since this allele was present in 51 % of the Brazilian (South American) samples.

Previous studies seemed to indicate that both SNP and amplification of *pvmdr1* are associated with variation in in vitro CQ susceptibility of *P. vivax* [17, 32]. It has been shown that the geometric mean of the CQ inhibitory concentration 50 % (IC50) was significantly higher in isolates carrying the Y976**F** mutation when compared to wild-type isolates in samples from Indonesia and Thailand [17]. However, the clear association between the clinical outcome following a three-day CQ treatment and non-synonymous mutations in this gene has never been demonstrated elsewhere. In fact, the single 976**F** mutant was not very common worldwide (7.4 %) [22], and the **F**F double mutant genotype was detected only in endemic regions of three countries: Brazil [36, 37], Honduras [38] and Papua New Guinea [39]. In the samples herein analysed, no significant difference was observed between the presence of double 976**F**/1076**L** mutant (12 %) and single 976**F** mutant (15 %) and no single 1076**L** mutant was noted. These findings suggest that polymorphisms at codons 976 and 1076 may not be strong indicators of CQ resistance since all *P. vivax* isolates were obtained from patients with good response to CQ therapy. In addition, 976**F** and 1076**L** mutants were also detected in *P. vivax* isolates in several countries in Africa and in South America from patients with no history of CQ recrudescence [23]. Probably, these mutations might have been introduced in these countries from Asia where these mutations are prevalent [23]. Interestingly, the 976**F** mutation in *P. vivax* isolates from Extra-Amazonian were not detected in areas where autochthonous malaria cases from Brazilian Atlantic Forest can occur. Thus, it seems that 976**F** mutations are more associated to geographical characteristics than to CQR.

Concerning codon 958, only samples from the Amazon Basin showed the **M**YF single mutant genotype, and double **M**Y**L** mutants were observed in Amazonas and Acre State isolates. On the other hand, all isolates from the Extra Amazon State of Rio de Janeiro had the double mutant 958+1076 (**M**Y**L**) genotype and these samples were wild type for codon 976. Once again, the heterogeneity in these *P. vivax* populations could reflect the genetic diversity rather than an association with CQR in endemic areas with different endemic profiles [6].

Considering that *pvmdr1* mutations should not be expected in CQ-sensitive parasites and that PQ efficacy is highly dependent on concurrent administration of a blood schizontocidal agent [40] and thus PQ could not circumvent CQR, the present findings seem to indicate that the *pvmdr1* gene is not a reliable marker of CQR.

## Conclusion

This study provides new data concerning the molecular characterization of *P. vivax* isolates from Brazilian Atlantic Forest. The SNP diversity observed in samples from New World is similar to those from Asia and Africa, and probably reflects a capacity for great functional variation, as already suggested [41]. Very little is known about the molecular mechanisms underlying drug resistance in *P. vivax* and most *loci* that have been suggested to be responsible for *P. vivax* CQR derived from orthologue *P. falciparum* drug CQR genes. Further investigations are needed to define a reliable molecular marker for monitoring CQR in the Brazilian *P. vivax* population.

## References

[1] Mendis K, Sina BJ, Marchesini P, Carter R (2001). The neglected burden of *Plasmodium vivax* malaria. Am J Trop Med Hyg.

[2] Battle KE, Gething PW, Elyazar IR, Moyes CL, Sinka ME, Howes RE (2012). The global public health significance of *Plasmodium vivax*. Adv Parasitol.

[3] Price RN, Tjitra E, Guerra CA, Yeung S, White NJ, Anstey NM (2007). Vivax malaria: neglected and not benign. Am J Trop Med Hyg.

[4] da Silva-Nunes M, Moreno M, Conn JE, Gamboa D, Abeles S, Vinetz JM (2012). Amazonian malaria: asymptomatic human reservoirs, diagnostic challenges, environmentally driven changes in mosquito vector populations, and the mandate for sustainable control strategies. Acta Trop.

[5] Oliveira-Ferreira J, Lacerda MV, Brasil P, Ladislau JL, Tauil PL, Daniel-Ribeiro CT (2010). Malaria in Brazil: an overview. Malar J..

[6] de Pina-Costa A, Brasil P, Di Santi SM, de Araujo MP, Suárez-Mutis MC, Santelli AC (2014). Malaria in Brazil: what happens outside the Amazonian endemic region. Mem Inst Oswaldo Cruz.

[7] Whitby M, Wood G, Veenendaal JR, Rieckmann K (1989). Chloroquine-resistant *Plasmodium vivax*. Lancet.

[8] Baird JK, Basri H, Purnomo Bangs MJ, Subianto B, Patchen LC (1991). Resistance to chloroquine by *Plasmodium vivax* in Irian Jaya. Indones. Am J Trop Med Hyg..

[9] Baird JK (2004). Chloroquine resistance in *Plasmodium vivax*. Antimicrob Agents Chemother.

[10] Lee KS, Kim TH, Kim ES, Lim HS, Yeom JS, Jun G, Park JW (2009). Chloroquine-resistant *Plasmodium vivax* in the Republic of Korea. Am J Trop Med Hyg.

[11] Gonçalves LA, Cravo P, Ferreira MU (2014). Emerging *Plasmodium vivax* resistance to chloroquine in South America: an overview. Mem Inst Oswaldo Cruz.

[12] Melo GC, Monteiro WM, Siqueira AM, Silva SR, Magalhães BML, Alencar ACC (2014). Expression levels of *pvcrt*-*o* and *pvmdr1* are associated with chloroquine resistance and severe *Plasmodium vivax* malaria in patients of the Brazilian Amazon. PLoS ONE.

[13] Alecrim MG, Alecrim W, Macêdo V (1999). *Plasmodium vivax* resistance to chloroquine (R2) and mefloquine (R3) in Brazilian Amazon region. Rev Soc Bras Med Trop.

[14] de Santana Filho FS, Arcanjo AR, Chehuan YM, Costa MR, Martinez-Espinosa FE, Vieira JL (2007). Chloroquine-resistant *Plasmodium vivax*. Brazilian Amazon. Emerg Infect Dis..

[15] Nomura T, Carlton JM, Baird JK, del Portillo HA, Fryauff DJ, Rathore D (2001). Evidence for different mechanisms of chloroquine resistance in 2 *Plasmodium* species that cause human malaria. J Infect Dis.

[16] Brega S, Meslin B, de Monbrison F, Severini C, Gradoni L, Udomsangpetch R (2005). Identification of the *Plasmodium vivax* mdr-like gene (*pvmdr1*) and analysis of single-nucleotide polymorphisms among isolates from different areas of endemicity. J Infect Dis.

[17] Suwanarusk R, Chavchich M, Russell B, Jaidee A, Chalfein F, Barends M (2008). Amplification of *pvmdr1* associated with multidrug-resistant *Plasmodium vivax*. J Infect Dis.

[18] Lu F, Lim CS, Nam DH, Kim K, Lin K, Kim TS, Lee HW (2011). Genetic polymorphism in *pvmdr1* and *pvcrt*-*o* genes in relation to in vitro drug susceptibility of *Plasmodium vivax* isolates from malaria-endemic countries. Acta Trop.

[19] Mint Lekweiry K, Ould Mohamed Salem Boukhary A, Gaillard T, Wurtz N, Bogreau H, Hafid JE (2012). Molecular surveillance of drug-resistant *Plasmodium vivax* using *pvdhfr*, *pvdhps* and *pvmdr1* markers in Nouakchott, Mauritania. J Antimicrob Chemother.

[20] Lin JT, Patel JC, Kharabora O, Sattabongkot J, Muth S, Ubalee R (2013). *Plasmodium vivax* isolates from Cambodia and Thailand show high genetic complexity and distinct patterns of *P. vivax* multidrug resistance gene 1 (*pvmdr1*) polymorphisms. Am J Trop Med Hyg.

[21] Ranjitkar S, Schousboe ML, Thomsen TT, Adhikari M, Kapel CMO, Bygbjerg IC (2011). Prevalence of molecular markers of anti-malarial drug resistance in *Plasmodium vivax* and *Plasmodium falciparum* in two districts of Nepal. Malar J..

[22] Shalini S, Chaudhuri S, Sutton PL, Mishra N, Srivastava N, David JK (2014). Chloroquine efficacy studies confirm drug susceptibility of *Plasmodium vivax* in Chennai. India Malar J..

[23] Vargas-Rodríguez R del C, Silva Bastos M da, Menezes MJ, Orjuela-Sánchez P, Ferreira MU (2012). Single-nucleotide polymorphism and copy number variation of the multidrug resistance-1 locus of *Plasmodium vivax*: local and global patterns. Am J Trop Med Hyg.

[24] Chehuan YF, Costa MRF, Costa JS, Alecrim MGC, Nogueira F, Silveira H (2013). *In vitro* chloroquine resistance for *Plasmodium vivax* isolates from the Western Brazilian Amazon. Malar J..

[25] Sá JM, Nomura T, Neves JDA, Baird JK, Wellems TE, Del Portillo HA (2005). *Plasmodium vivax*: allele variants of the *mdr1* gene do not associate with chloroquine resistance among isolates from Brazil, Papua, and monkey-adapted strains. Exp Parasitol.

[26] Marques MM, Santana Costa MRF, Filho FS, Vieira JLF, Nascimento MTS, Brasil LW (2014). *Plasmodium vivax* chloroquine resistance and anemia in the Western Brazilian Amazon. Antimicrobial Agents Chemother..

[27] Aguiar AC, Pereira DB, Amaral NS, De Marco L, Krettli AU (2014). *Plasmodium vivax* and *Plasmodium falciparum* ex vivo susceptibility to anti-malarials and gene characterization in Rondônia, West Amazon. Brazil. Malar J..

[28] Torres KL, Figueiredo DV, Zalis MG, Daniel-Ribeiro CT, Alecrim W, de Ferreira-da-Cruz M (2006). F. Standardization of a very specific and sensitive single PCR for detection of *Plasmodium* *vivax* in low parasitized individuals and its usefulness for screening blood donors. Parasitol Res.

[29] Brega S, de Monbrison F, Severini C, Udomsangpetch R, Sutanto I, Ruckert P (2004). Real-time PCR for dihydrofolate reductase gene single-nucleotide polymorphisms in *Plasmodium vivax* isolates. Antimicrob Agents Chemother.

[30] Otto TD, Vasconcellos EA, Gomes LH, Moreira AS, Degrave WM, Mendonça-Lima L (2008). ChromaPipe: a pipeline for analysis, quality control and management for a DNA sequencing facility. Genet Mol Res.

[31] Añez A, Moscoso M, Laguna Á, Garnica C, Melgar V, Cuba M (2015). Resistance of infection by *Plasmodium vivax* to chloroquine in Bolivia. Malar J..

[32] Fernández-Becerra C, Pinazo MJ, González A, Alonso PL, del Portillo HA, Gascón J (2009). Increased expression levels of the *pvcrt*-*o* and *pvmdr1* genes in a patient with severe *Plasmodium vivax* malaria. Malar J..

[33] Barnadas C, Ratsimbasoa A, Tichit M, Bouchier C, Jahevitra M, Picot S (2008). *Plasmodium* *vivax* resistance to chloroquine in Madagascar: clinical efficacy and polymorphisms in *pvmdr1* and *pvcrt*-*o* genes. Antimicrob Agents Chemother.

[34] Rungsihirunrat K, Muhamad P, Chaijaroenkul W, Kuesap J, Na-Bangchang K (2015). *Plasmodium vivax* drug resistance genes; *Pvmdr1* and *Pvcrt*-*o* polymorphisms in relation to chloroquine sensitivity from a malaria endemic area of Thailand. Korean J Parasitol.

[35] Schousboe ML, Ranjitkar S, Rajakaruna RS, Amerasinghe PH, Morales F, Pearce R (2015). Multiple origins of mutations in the mdr1 gene: a putative marker of chloroquine resistance in *P. vivax*. PLoS Negl Trop Dis..

[36] Orjuela-Sánchez P, de Santana Filho FS, Machado-Lima A, Chehuan YF, Costa MR, Alecrim MD (2009). Analysis of single-nucleotide polymorphisms in the crt-o and mdr1 genes of *Plasmodium vivax* among chloroquine-resistant isolates from the Brazilian Amazon region. Antimicrob Agents Chemother.

[37] Gama BE, Oliveira NK, Souza JM, Daniel-Ribeiro CT, de Ferreira-da-Cruz M (2009). F. Characterisation of *pvmdr1* and *pvdhfr* genes associated with chemoresistance in Brazilian *Plasmodium vivax* isolates. Mem Inst Oswaldo Cruz.

[38] Jovel IT, Mejía RE, Banegas E, Piedade R, Alger J, Fontecha G (2011). Drug resistance associated genetic polymorphisms in *Plasmodium falciparum* and *Plasmodium vivax* collected in Honduras. Cent Am. Malar J..

[39] Marfurt J, de Monbrison F, Brega S, Barbollat L, Müller I, Sie A, Goroti M (2008). Molecular markers of in vivo *Plasmodium vivax* resistance to amodiaquine plus sulfadoxine-pyrimethamine: mutations in *pvdhfr* and *pvmdr1*. J Infect Dis.

[40] Fernando D, Rodrigo C, Rajapakse S (2011). Primaquine in vivax malaria: an update and review on management issues. Malar J..

[41] Neafsey DE, Galinsky K, Jiang RH, Young L, Sykes SM, Saif S, Gujja S (2012). The malaria parasite *Plamodium vivax* exhibits greater genetic diversity than *Plasmodium falciparum*. Nat Genet.

